# Acute myocardial infarction due to coronary embolism caused by a metastatic mass from lung cancer

**DOI:** 10.1186/s12872-023-03505-3

**Published:** 2023-09-15

**Authors:** Yingli Zhao, Meijiao Mao, Na Zhang, Shuai Zhang, Wangkang Niku, Ling Zhu, Xiujuan Shi, Zhaoyi Yang, Yanwen Wang, Bing Deng, Wang Zheng

**Affiliations:** Department of Cardiology, Longhua Hospital, Shanghai University of Traditional Chinese Medicine, Shanghai, 200032 China

**Keywords:** Acute myocardial infarction, Lung tumor, Coronary embolism

## Abstract

**Background:**

Acute arterial embolism due to tumor embolus is a rare complication in cancer patients, even rarer is lung tumor embolization leading to acute myocardial infarction. We report a patient who had a diagnosis of acute myocardial infarction(AMI)which was brought on by a coronary artery embolism by a metastatic lung cancer tumor. Clinicians need to be aware that tumor embolism can result in AMI.

**Case presentation:**

An 80-yeal-old male patient presented with persistent chest pain for 2 h and his electrocardiogram(ECG)showed anterior ST-segment elevation myocardial infarction. Instead of implanting a stent, thrombus aspiration was performed. Pathological examination of coronary artery thrombosis showed that a few sporadic atypical epithelial cells were scattered in the thrombus-like tissue. Combined with immune phenotype and clinical history, metastatic squamous cell carcinoma is more likely.

**Conclusions:**

We report a rare case of a patient who was diagnosed of AMI due to a coronary artery embolism by a metastatic mass from lung cancer. Since there is no evidence-based protocol available for the treatment of isolated coronary thrombosis, we used thrombus aspiration to treat thrombosis rather than implanting a stent.

## Background

Acute myocardial infarction (AMI) is very common in clinics and usually caused by coronary plaque rupture, erosion, or nodules, however there are also unusual causes [1]. Coronary embolism(CE), a rare cause of AMI, refers to the entry of obstructive substances into coronary arteries, block their blood flow and result in ischemia. The incidence of AMI caused by CE is 2.9%. Acute arterial embolism caused by tumor embolism is a very uncommon complication in cancer patients [2], and AMI caused by pulmonary tumor embolism is even rarest. It has previously been reported that lung tumor embolism occurs in the coronary artery [3–5]. We report a case of AMI caused by lung cancer emboli.

## Case presentation

**Fig. 1:**
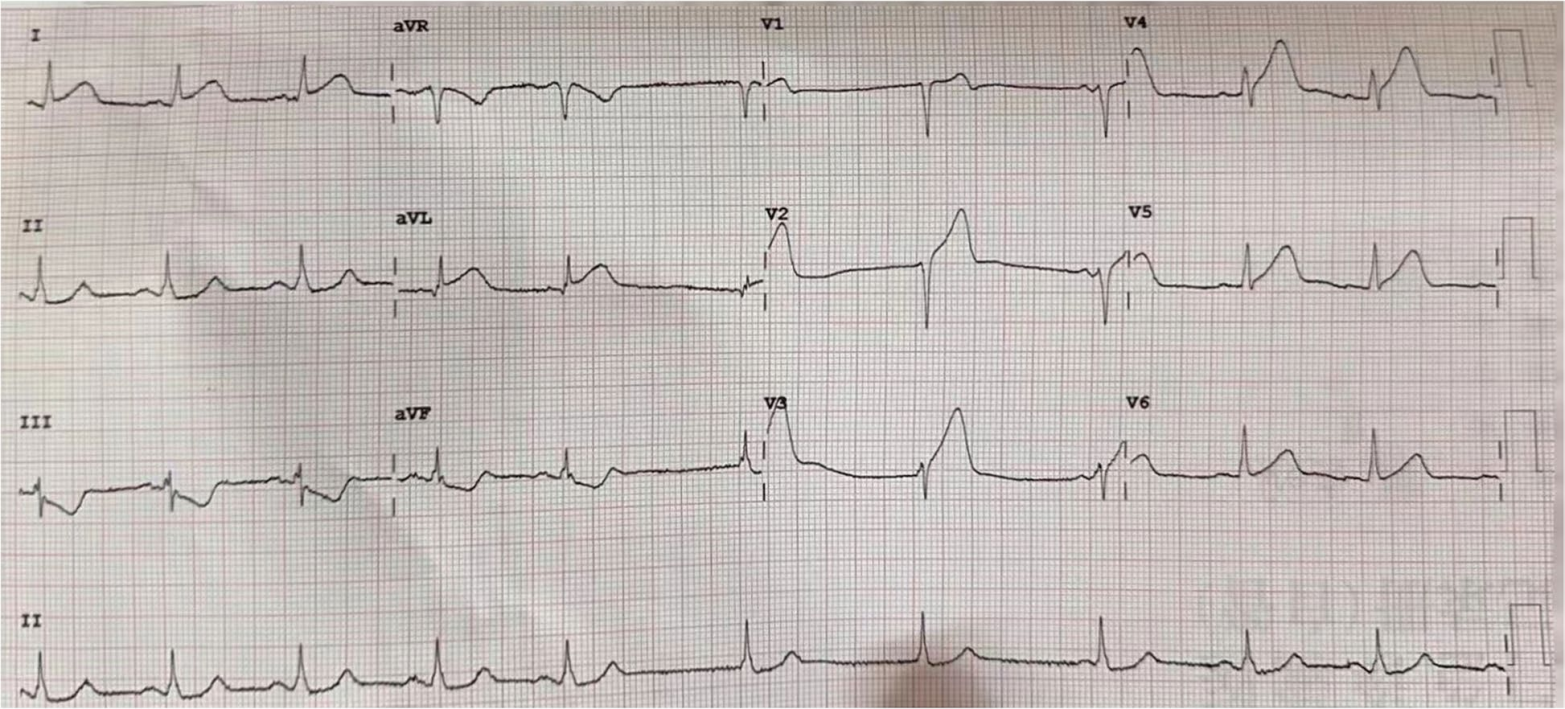
Electrocardiogram showing ST-elevation in anterior leads

**Fig. 2:**
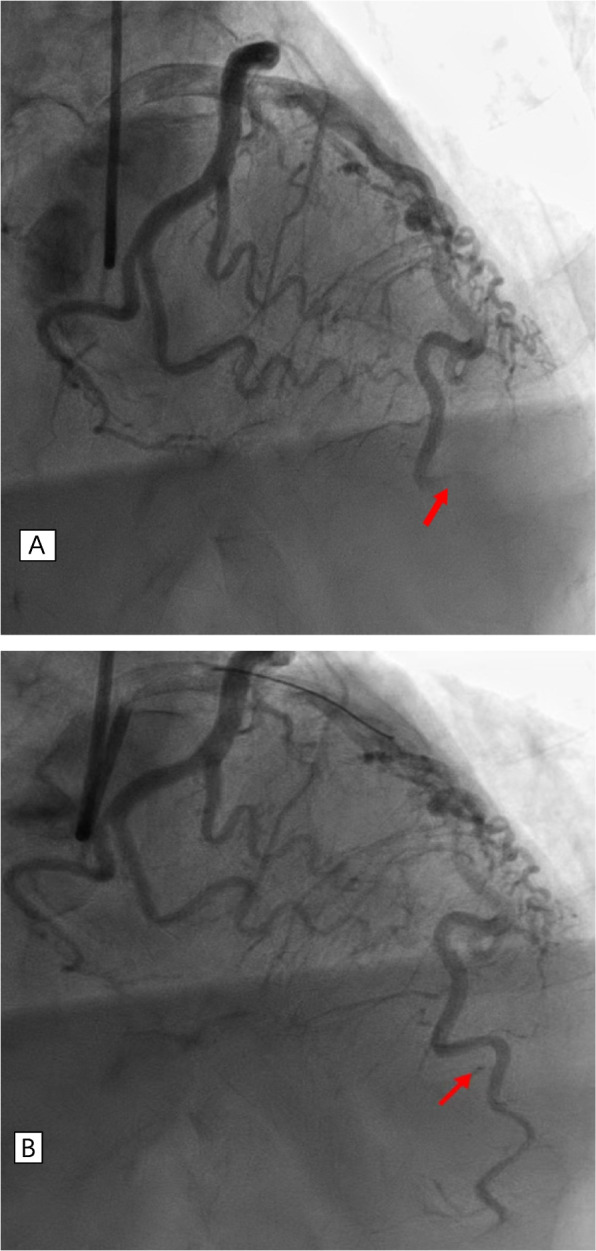
Coronary angiogram of left anterior descending coronary artery. **A** the distal of the anterior descending was completely occluded. **B** The distal of the anterior descending was even and no obvious stenosis was observed after the thrombotic suction

**Fig. 3:**
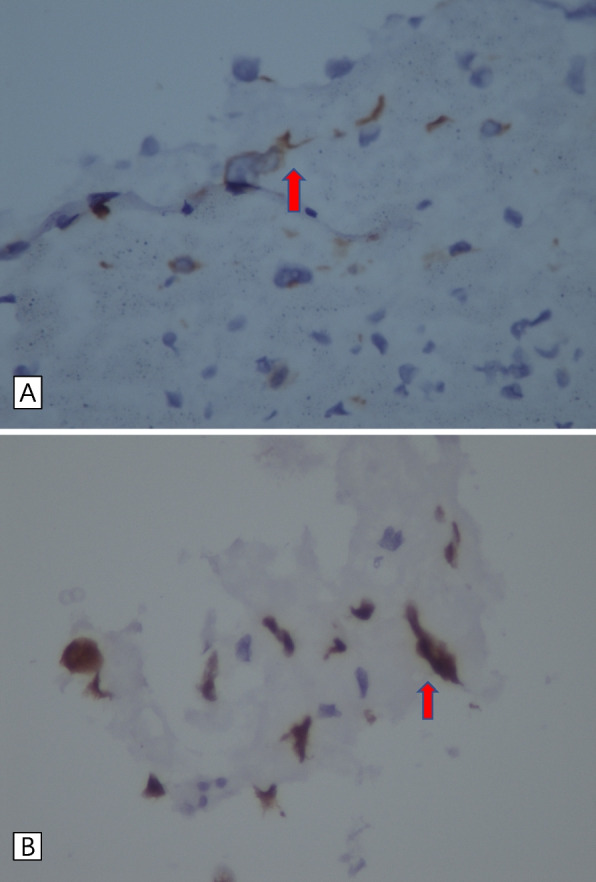
Left anterior descending coronary artery occluded by squamous cell carcinoma. **A** CK7 was highly expressed in cancer thrombus cells by immunohistochemical staining, **B** P40 was highly expressed in cancer thrombus cells by immunohistochemical staining

An 80-yeal-old male patient presented with persistent chest pain for 2 h and his electrocardiogram(ECG)showed anterior ST-segment elevation myocardial infarction (Fig. 1). Emergency coronary angiography was performed and the results showed the distal of the anterior descending was completely occluded and a significant amount of red and white thrombus was removed by using a thrombotic suction (Fig. 2A, B). The distal of the anterior descending was even and no obvious stenosis was observed and TIMI blood flow was grade 3, so we did not implant the stent. Echocardiography revealed normal overall left ventricular systolic function while there was no sign of a left ventricular thrombus or patent foramen ovale. The patient had a history of pulmonary space occupying for 1 year and we performed a histological examination of the thrombus. Pathological examination of coronary artery thrombosis showed that a small amount of atypical epithelial cells were scattered in the thrombus like tissue. Combined with immune phenotype and clinical history, metastatic squamous cell carcinoma is more likely. Immunohistochemistry results revealed that: A: AE1/AE3 (+), TTF-1 (-) P40 (+), CK7 (+), CK20 (-), Villin(-) (Fig. 3A, B)

## Discussion

Left atrial myxoma, atrial fibrillation, cardiomyopathy, valvular heart disease, malignancy, infective endocarditis, and atrial septal defect may cause CE induced AMI. Mert I Hayiroglu reported an 18-year-old boy who used marijuana was admitted to the hospital with severe substernal chest pain and coronary angiography revealed a considerable mid-segment blockage of the left anterior descending artery with a recent thrombus [6]. Mert İlker Hayıroğlu reported a 74-year-old man who was also experiencing dyspnea and excruciating chest discomfort arrived to the emergency room. The coronary angiography revealed that the proximal left anterior descending artery was completely blocked. Deep venous thrombosis was detected bilaterally by venous doppler ultrasonography. Biatrial thrombus development connected by the patent foramen ovale was detected by transthoracic echocardiography [7]. In our situation, the patient did not use any illicit drugs or materials, nor did he have any risk factors for coronary heart disease, such as hypertension, diabetes, hyperlipidemia or obesity. The incidence of CE in the left anterior descending branch, left circumflex branch, and right coronary artery dose not significantly differ, per the data that are now available [8]. The CHA2DS2-VASc score is generally used to acess the thromboembolism risk in non-valvular atrial fibrillation patients and Tufan evaluated its value in acute coronary syndromes [9]. However, more studies are requeired to further corroborate this.

Malignant tumors lead to venous or arterial thrombosis due to abnormal clotting, platelet activation, and endothelial dysfunction [10]. According to reports, 4.5% of patients develop venous thromboembolism and 1.5% experience arterial thromboembolism. Lung cancer is the most typical original malignant tumor that spreads to the heart [11]. Lung cancer invades the heart in two different ways: directly from the primary tumor or metastatic lymph nodes, and continuously through the pulmonary veins. Most arterial tumor embolism occurs during or shortly after pulmonary resection, rather than spontaneously as in this case [12]. To our knowledge, spontaneous global tumor embolism occurs in very few cases where the tumor invades the pulmonary veins of lung cancer, and the prognosis of most patients is poor [13, 14]. However, clinicians should be aware that tumor embolism is a potential cause of AMI. Acute arterial embolism caused by tumor embolus is a rare complication in tumor patients. Following the tumor nests’ invasion of the pulmonary veins, ejection from the body is the acknowledged mechanism of arterial embolization in malignant lung tumors [2]. In contrast to the right atrium and the two ventricles, the left atrium is anatomically a structure connected to the hilum of the lung by pulmonary veins, which explains why it is most frequently directly attacked by central lung tumors [15]. TTF-1 is expressed in 75-85% of lung adenocarcinoma, but not in squamous cell carcinoma, so it is mainly utilized to distinguish adenocarcinoma from squamous cell carcinoma. CK7 has a high sensitivity and is expressed in almost 100% of lung adenocarcinoma, but its specificity is poor and it is also expressed in 30–70% of lung squamous cell carcinoma, and P40 is basically not expressed in lung adenocarcinoma, but is expressed in more than 96.8% of squamous cell carcinoma. In our case, the patient had TTF-1 (-), CK7 (+), and P40 (+), so metastatic squamous cell carcinoma was highly likely [16].

 Other arterial systems, such as the aorta, femoral artery, limb artery, and mesenteric artery, can also experience lung tumor embolism [17]. Aortic bifurcation or femoral vessels (50%) and cerebral circulation (30%) are the locations of tumor embolization that occur most frequently [18]. Advanced primary or metastatic lung cancers frequently pass via the pulmonary veins and reach the arterial system [19, 20].

 Regarding the management of coronary embolization, there is currently no agreement. There are currently two therapies available for early ST-segment elevation AMI: intravenous thrombolysis and percutaneous intervention. For this kind of cardiac embolism, intravenous thrombolysis has been suggested in the literature [21–24]. Dual dose thrombolytic therapy has been demonstrated in certain studies to be superior to single dose therapy [25], however this is still up for debate. Thrombolytic therapy is not necessary if the dislodged embolus is an infected plant [26]. There have been reports of aspiration being used for intra-coronary embolization [27–29].

## Conclusion

We described a rare case of a patient who had an AMI diagnosis brought on by a coronary artery embolism by a metastatic mass from lung cancer. Since there is no evidence-based protocol for the treatment of isolated coronary thrombosis we used thrombus aspiration to deal with thrombosis rather than implanting a stent. However, there was a flaw in our case because intravascular ultrasound(IVUS)or optical coherence tomography (OCT) were not to be used to evaluate the lesion’s susceptibility and composition.

## Data Availability

The data in this case report are not publicly available due to the privacy policies of the hospital but may be requested from the corresponding author if deemed reasonable.

## Abbreviations

AMI: Acute myocardial infarction
CE: Coronary embolism
ECG: Electrocardiogram
IVUS: Intravascular ultrasound
OCT: Optical coherence tomography
